# A Case of an 80-Year-Old Woman With Chronic Eosinophilic Pneumonia and Clinically Significant Giant Hepatic Hemangioma

**DOI:** 10.7759/cureus.98917

**Published:** 2025-12-10

**Authors:** Vicken Khazar, Samuel Escobar, Amy Huang, Anthony M Szema

**Affiliations:** 1 Medicine, Donald and Barbara Zucker School of Medicine at Hofstra/Northwell, Hempstead, USA; 2 Allergy and Immunology, Warren Alpert Medical School of Brown University, Providence, USA; 3 Allergy and Immunology, Three Village Allergy and Asthma, PLLC, Centereach, USA; 4 Allergy and Immunology, Donald and Barbara Zucker School of Medicine at Hofstra/Northwell, Hempstead, USA

**Keywords:** chronic eosinophilic pneumonia, giant hepatic hemangioma, hepatology, medical management of hepatic hemangioma, rare benign hepatic tumor

## Abstract

An 80-year-old woman with a history of chronic eosinophilic pneumonia (CEP), severe persistent asthma, concomitant severe idiopathic urticaria, fibrous dysplasia of bone, osteoporosis resulting from years of oral corticosteroid usage, and arthralgia, presented to the allergy and immunology clinic with abdominal discomfort. Abdominal and pelvic CT scan showed a hepatic hemangioma, measuring approximately 8.2 x 6.4 cm. The patient underwent arterial embolization with no improvement in size or symptoms. CT scan of the same area taken eight years later had the same large, well-defined hypodense lesion in the right hepatic lobe that increased in size to 10.5 x 7.3 cm. The larger hemangioma now resulted in a mass effect on the upper pole of the right kidney, encroaching on the second portion of the duodenum, and pushing the vertebral column to the left. The patient’s CEP was well controlled after desensitization to reslizumab, which initially induced urticaria. Her asthma symptoms are well controlled with reslizumab.

Our patient demonstrates that, even in older age groups, it is still possible to present with mega hepatic hemangiomas, despite the median age of presentation being 46. Patients who experience abdominal symptoms, increased hemangioma size, and anxiety frequently undergo surgical resection. Our patient also met the criteria for a resection. The patient was referred to the University of Pittsburgh for excision. However, despite her CEP and asthma under control, following the principle of primum non nocere (first, do no harm), no treatment was advised, opting for close observation. While larger hemangiomas have been reported, this case demonstrates that even in the elderly, with multiple co-morbidities, one may tolerate a growing mega hepatic hemangioma. Age and comorbidity may preclude operative therapy.

## Introduction

Hepatic hemangiomas (HH) are thought to be vascular malformations characterized by disorganized cavity enlargement via dilation, as opposed to hyperplasia or hypertrophy, and result in abnormal growth of blood vessels within the liver. They are the most common benign hepatic tumor at encompassing 20% of cases, typically present in individuals ages 30-50 years, and are more common in women than men [1-2].

While most HHs are small and benign, these tumors are considered “giant” when their diameter exceeds 5 cm and “enormous” when greater than 15cm. Hemangiomas are most commonly asymptomatic and are found incidentally on unrelated radiologic imaging, falling in the tumor category of “incidentalomas,” with a median age of presentation at 46 years old [1,3]. When giant hemangiomas present symptomatically, it is most often with abdominal pain or discomfort, early satiety, nausea, dyspnea, and postprandial bloating. Symptomatic HH warrants further workup for informed management [3].

Although the etiology of HH formation and progression is poorly understood, their enlargement has been associated with increased exposure to estrogen and progesterone, as in the setting of hormone replacement therapy (HRT), hormonal contraceptive use, and pregnancy. Given this, HHs are still seen in those without increased exposure to estrogen and progesterone, such as those not taking HRT or, as in our patient’s case, post-menopausal [1,4]. Despite being a relatively non-rare benign tumor, there is considerable interest in characterizing the epidemiology, progression, and modes of management of HHs given their unpredictable growth patterns, often vague symptoms, and potential for complications [5].

Chronic eosinophilic pneumonia (CEP) is a rare interstitial lung disease defined by marked eosinophilic infiltration of the alveolar spaces and pulmonary interstitium, leading to chronic pulmonary inflammation. Patients often develop symptoms over weeks to months, including progressive dyspnea, cough (often non-productive), low-grade fever, night sweats, weight loss, fatigue, and sometimes wheezing - though a history of asthma or atopy is common but not required [6]. On imaging, CEP typically manifests as bilateral peripheral or subpleural consolidations and/or ground-glass opacities on high-resolution CT or chest radiograph - sometimes described as a “photographic negative of pulmonary edema [7]." Bronchoalveolar lavage (BAL) frequently demonstrates marked eosinophilia (often ≥ 40 % of cells), which, in the correct clinical and radiologic context, may obviate the need for lung biopsy [8]. Treatment with systemic corticosteroids usually results in rapid clinical and radiographic improvement, often within days to a few weeks. However, relapse is common: long-term follow-up studies indicate that a substantial proportion of patients (≈ 50-60 %) require prolonged low-dose maintenance therapy to prevent recurrence. Over time - particularly in patients with repeated relapses - some may develop persistent impairment in pulmonary function, such as reduced diffusing capacity (DLCO) or airflow obstruction, underscoring the importance of long-term monitoring [9].

## Case presentation

Our patient is an 80-year-old woman with a history of CEP, severe persistent asthma, severe idiopathic urticaria, fibrous bone dysplasia, arthralgia, osteoporosis, and resected papillary thyroid carcinoma who presented to the clinic with abdominal discomfort, early satiety, and increased dyspnea on exertion. 

**Figure 1:**
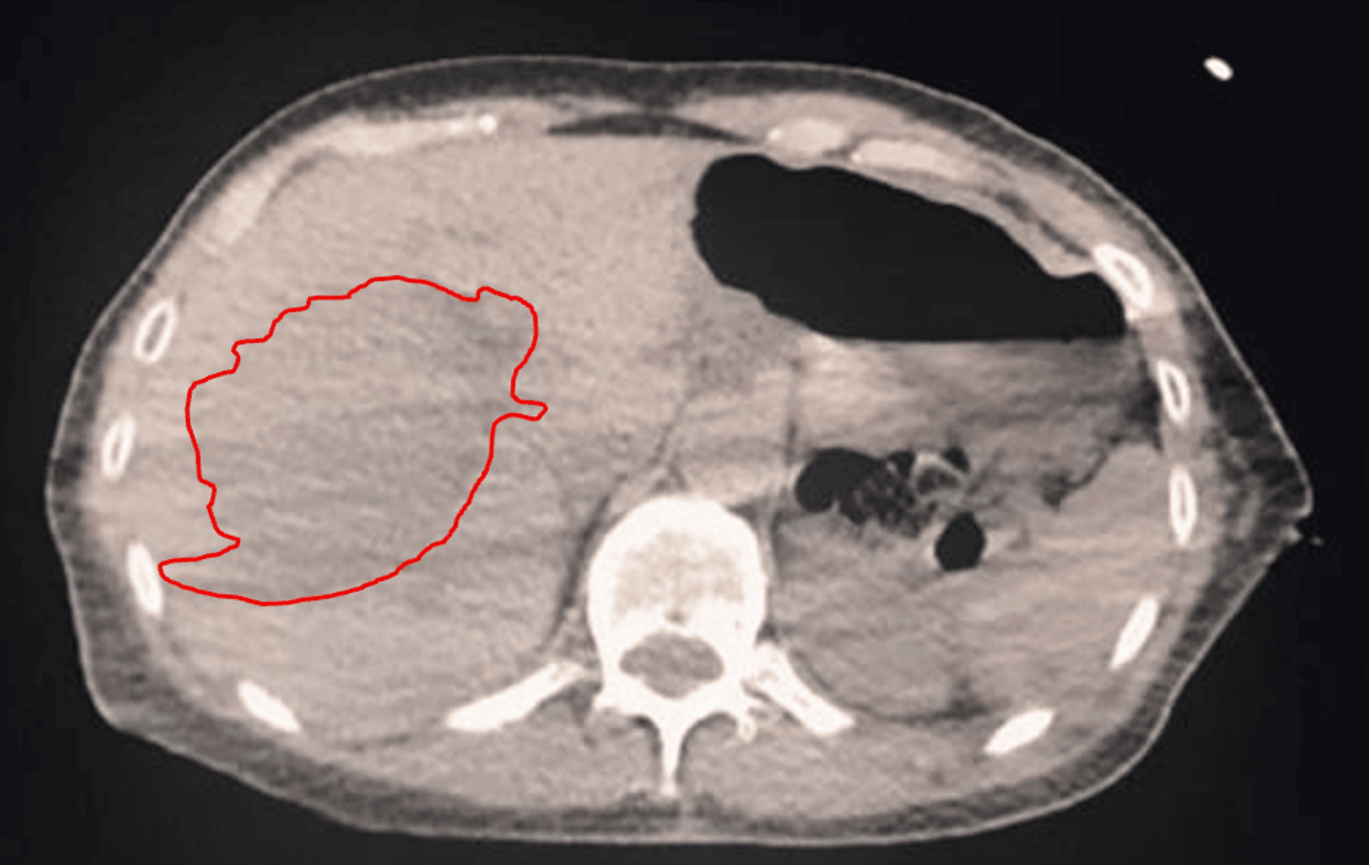
Initial axial CT of the abdomen demonstrates a hemangioma (red tracing) measuring 8.2 x 6.4 cm.

**Figure 2:**
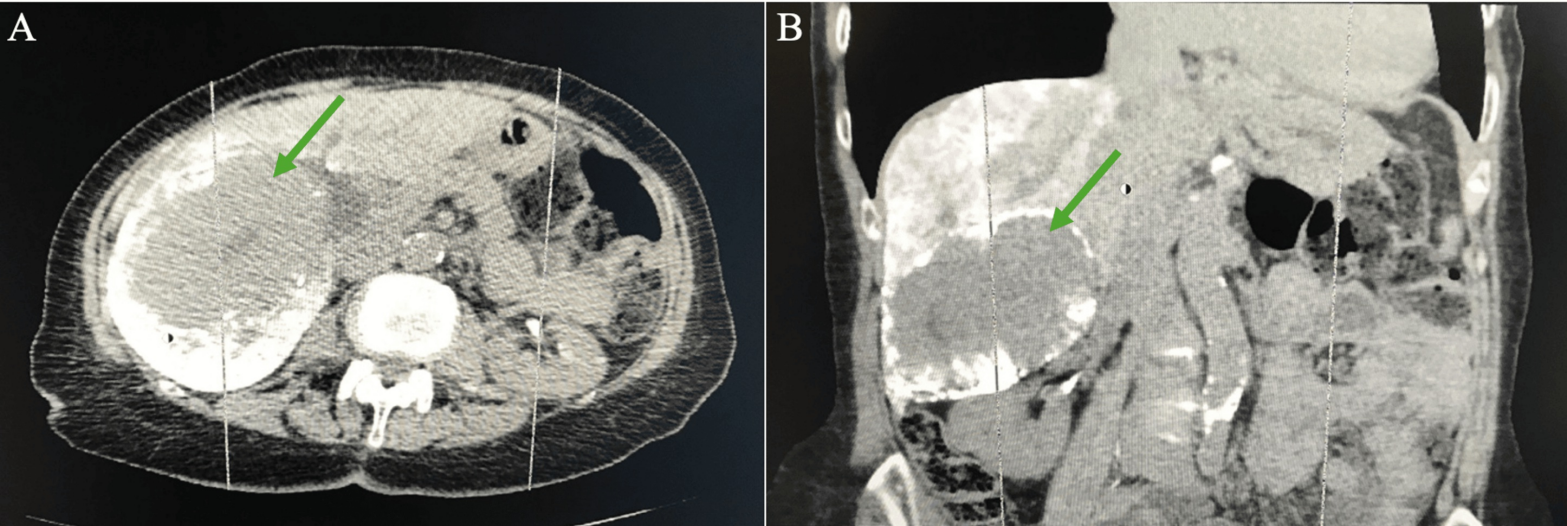
CT of abdomen with transverse (A) and sagittal (B) views taken after liquid embolization with lipiodol and bleomycin, 8 years after initial CT. Hepatic hemangioma (green arrow) measures approximately 10.3 x 7.3 cm.

Initial abdominal CT scans from 2018 showed a hepatic hemangioma measuring approximately 8.2 x 6.4 cm (Figure 1). She underwent arterial embolization with no improvement in size or symptoms. Follow-up CT scan of the same area taken eight years later demonstrated that the same large well-defined hypodense lesion in the right hepatic lobe had increased in size to 10.5 x 7.3 cm (Figure 2). The hemangioma now resulted in a mass effect on the upper pole of the right kidney, encroaching on the second portion of the duodenum, and pushing the vertebral column to the left. Her transaminase testing was within normal limits with aspartate aminotransferase (AST) and alanine aminotransferase (ALT) levels at 15 IU/L (4-40 IU/L) and 9 IU/L (4-41 IU/L), respectively.

**Figure 3:**
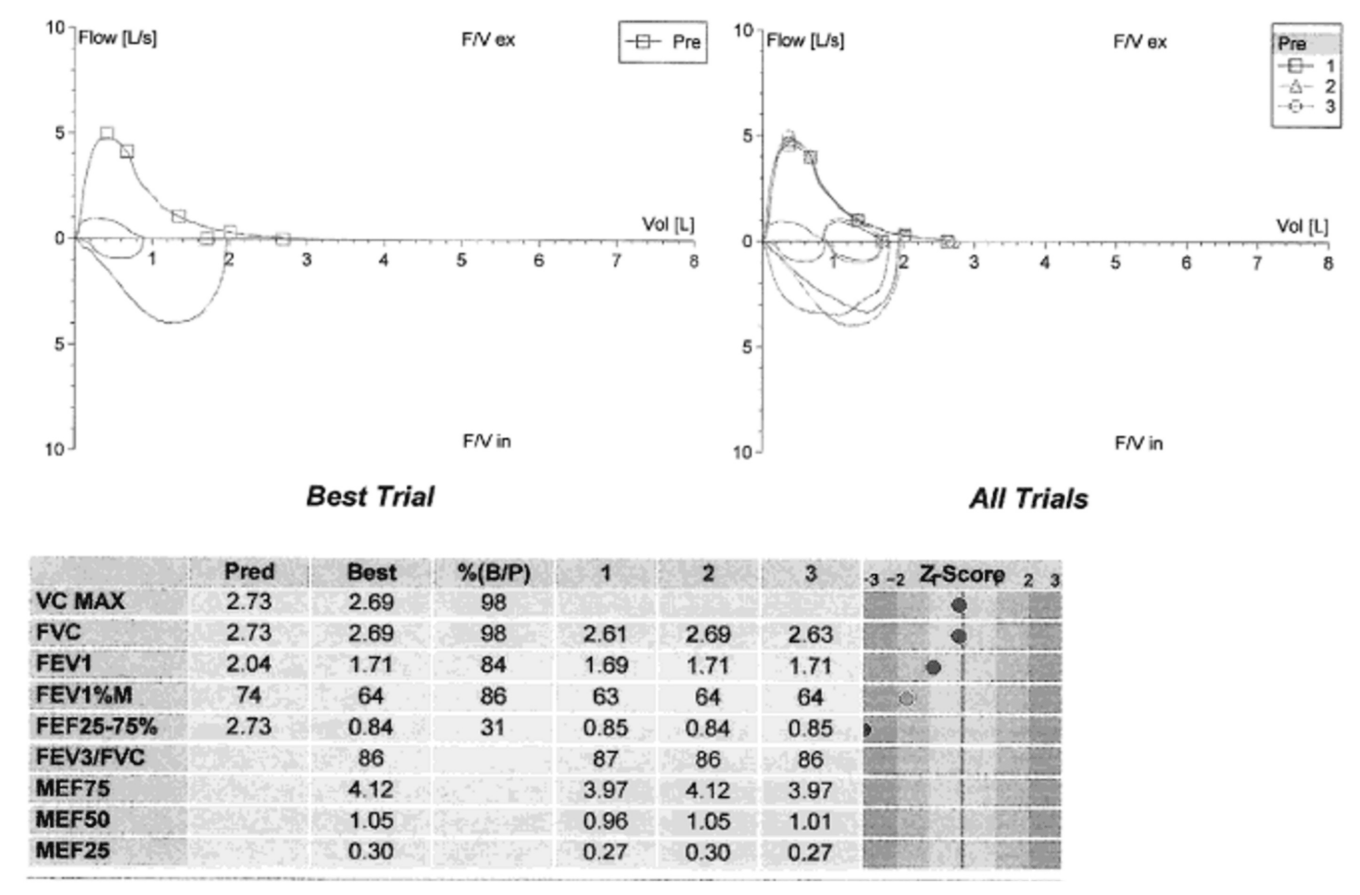
Spirometry indicates an obstructive ventilatory defect FVC: Forced vital capacity; FEV1: Forced expiratory volume in 1 second; FEF: Forced expiratory flow

Although her CEP and asthma have been well controlled with reslizumab, when she presented with her new onset abdominal discomfort, early satiety, and dyspnea on exertion, her spirometry results indicated worsened obstructive ventilatory defect despite her having normal lung volumes and diffusing capacity (Figure 3).

## Discussion

Surgical intervention is common among patients presenting with a giant hepatic hemangioma to prevent the possibility of spontaneous rupture, resulting in peritoneal cavity hemorrhage. Among 27 cases of spontaneous hepatic hemangioma rupture, 22 patients (95.7%) underwent surgery [4].

A multidisciplinary team met to discuss this patient’s management. Our patient was offered various treatment options, including surgical intervention. The patient opted for conservative treatment and declined surgery.

Our patient continued to physically and emotionally struggle with the symptoms resulting from the giant hemangioma, leading her to again seek medical management. Another multidisciplinary team convened to discuss viable treatments, which were then offered to the patient. This time, she decided to pursue a more conservative interventional radiology procedure.

She underwent liquid embolization with lipiodol and bleomycin. Though the hemangioma is largely the same size as prior to embolization, measuring approximately 10.3 x 7.3 cm, our patient endorsed resolution of early satiety, abdominal discomfort, and dyspnea on exertion back to baseline. This clinical response highlights that symptomatic relief may not correlate directly with changes in tumor size, particularly when mass effect rather than total lesion volume is the primary driver of symptoms. Embolization may reduce internal vascularity and pressure within the lesion, alleviating local compression of adjacent structures even without a substantial decrease in overall dimensions, for instance. As demonstrated in this case, meaningful improvement in quality of life can therefore be achieved through targeted intervention despite radiologic stability, emphasizing the importance of individualized assessment of symptom burden when considering treatment success.

This is an unusual case of a patient presenting with hepatic hemangioma and CEP, and it is especially rare to see large hepatic hemangiomas that continue to enlarge in the geriatric population. Although chronic eosinophilic disease and vascular malformations may coexist, current evidence does not support a mechanistic association between hepatic hemangiomas and chronic eosinophilic pneumonia or other eosinophilic inflammatory states. Hepatic hemangiomas are frequently encountered as incidental findings during the evaluation of unrelated conditions, reflecting their high prevalence rather than a shared pathological process. Their presence alongside complex diseases can inadvertently lead clinicians to pursue a unifying diagnosis despite the likelihood of independent processes. Recognizing HHs as common incidentalomas is therefore critical in preventing diagnostic overreach and ensuring that clinical decision-making remains grounded in evidence-based assessment rather than coincidental coexistence of findings.

## Conclusions

Ockham’s razor, which states numquam ponenda est pluralitas sine necessitate (plurality must never be posited without necessity), has been a valuable teaching tool used by healthcare providers to make a unifying diagnosis, but it cannot be applied to every situation. Currently, there is no connection between CEP and hepatic hemangiomas.

However, with further testing, such as a liver biopsy and RNA sequencing, a link may surface. In the meantime, open communication, respecting patients’ decisions, providing emotional support, as well as utilizing a multidisciplinary approach are essential to provide individualized, quality care.
